# Current Practice and Barriers to the Implementation of Mobilization in ICUs in Japan: A Multicenter Prospective Cohort Study

**DOI:** 10.3390/jcm12123955

**Published:** 2023-06-09

**Authors:** Hideaki Sakuramoto, Kensuke Nakamura, Akira Ouchi, Saiko Okamoto, Shinichi Watanabe, Keibun Liu, Yasunari Morita, Hajime Katsukawa, Toru Kotani

**Affiliations:** 1 Department of Critical Care and Disaster Nursing, Japanese Red Cross Kyushu International College of Nursing, Munakata 811-4157, Fukuoka, Japan; gongehead@yahoo.co.jp; 2 Department of Critical Care Medicine, Yokohama City University Hospital, 3-9 Fukuura, Kanazawa-ku, Yokohama 236-0004, Kanagawa, Japan; 3 Department of Emergency and Critical Care Medicine, Hitachi General Hospital, 2-1-1 Jonancho, Hitachi 317-0077, Ibaraki, Japan; chara.ponpoco@gmail.com; 4 Department of Adult Health Nursing, College of Nursing, Ibaraki Christian University, Hitachi 319-1295, Ibaraki, Japan; akira1q85@gmail.com; 5 Department of Physical Therapy, Faculty of Rehabilitation, Gifu University of Health Science, 2-92 Higashiuzura, Gifu 500-8281, Gifu, Japan; billabonghonor@yahoo.co.jp; 6 Critical Care Research Group, The Prince Charles Hospital, 627 Rode Rd, Chermside, QLD 4032, Australia; keiliu0406@gmail.com; 7 Faculty of Medicine, The University of Queensland, 20 Weightman St, Herston, QLD 4006, Australia; 8 Non-Profit Organization ICU Collaboration Network (ICON), 2-15-13 Hongo, Bunkyo-ku, Tokyo 113-0033, Japan; 9 Department of Emergency and Intensive Care Medicine, National Hospital Organization Nagoya Medical Center, Nagoya 460-0001, Aichi, Japan; moltlyme2@yahoo.co.jp; 10 Japanese Society for Early Mobilization, 1-2-12 Kudankita, Chiyoda-ku, Tokyo 102-0073, Japan; winegood21@gmail.com; 11 Department of Intensive Care Medicine, School of Medicine, Showa University, 1-5-8 Hatanodai, Shinagawa-ku, Tokyo 142-8666, Japan; trkotani@med.showa-u.ac.jp

**Keywords:** mobilization, barrier, critical care, intensive care

## Abstract

**Background**: Limited information is currently available on the barriers to implementing mobilization at the bedside for critically ill patients. Therefore, we investigated the current practice of and barriers to the implementation of mobilization in intensive care units (ICU). **Methods**: A multicenter prospective observational study was conducted at nine hospitals between June 2019 and December 2019. Consecutive patients admitted to the ICU for more than 48 h were enrolled. Quantitative data were analyzed descriptively, and qualitative data were analyzed thematically. **Results**: The 203 patients enrolled in the present study were divided into 69 elective surgical patients and 134 unplanned admission patients. The mean periods of time until the initiation of rehabilitation programs after ICU admission were 2.9 ± 7.7 and 1.7 ± 2.0 days, respectively. Median ICU mobility scales were five (Interquartile range: three and eight) and six (Interquartile range: three and nine), respectively. The most common barriers to mobilization in the ICU were circulatory instability (29.9%) and a physician’s order for postoperative bed rest (23.4%) in the unplanned admission and elective surgery groups, respectively. **Conclusions**: Rehabilitation programs were initiated later for unplanned admission patients and were less intense than those for elective surgical patients, irrespective of the time after ICU admission.

## 1. Introduction

Physical function and quality of life are impaired in critically ill patients following their discharge from the intensive care unit (ICU) [1]. Loss of muscle strength was previously reported in 25–50% of patients discharged from the ICU [2]. As a countermeasure, mobilization, such as walking, is performed by critically ill patients. The mobilization of critically ill patients is considered to be beneficial for improving physical function and shortening not only the length of mechanical ventilation, but also the length of stay in the hospital [3,4].

However, there are several barriers to implementing the mobilization of critically ill patients, which are based on patient and provider factors [5,6,7]. Provider factors include a lack of knowledge of the benefits of mobilization, the unavailability of professionals and time to mobilize patients, and the lack of training for health care providers, such as intensivists, nurses, and physiotherapists [5,6,7]. Specifically, more than half of the physical therapists and nurses in the previous study identified limited staffing to routinely mobilize patients as a barrier on the part of the health care providers [5,7]. Patient factors have been reported to include excessive sedation, delirium, the risk of musculoskeletal self-injury, excessive stress at mobilization, and clinical instability, such as hypotension and hypoxia [5,7]. In particular, medical instability and the risk of dislodging devices or lines were cited as barriers by more than half of the providers in prior studies [5,7].

Most of these studies conducted questionnaire surveys or interviews of health care providers and did not investigate the reasons why mobilization was not performed at the actual bedside [5,7,8]. Therefore, the types of barriers that are commonly encountered remain unclear [5,7,8]. Based on these findings, the present study focused on the clinical practice of and barriers to the mobilization of critically ill patients. The aim of this study was to identify the current practice of and barriers to the implementation of mobilization, defined as a rehabilitation level of sitting at the edge of the bed or higher, in Japanese ICUs.

## 2. Materials and Methods

### 2.1. Study Design

A multicenter prospective observational study was conducted at nine hospitals between June 2019 and December 2019. This study was approved by the Ethics Committees of Nagoya Medical Center (2018093) and the eight other participating hospitals (Hitachi General Hospital, Nagasaki University Hospital, Fukuyama City Hospital, Naha City Hospital, Yuuai Medical Center, Tokushukai General Hospital, Showa University Hospital, and Tokyo Women’s Medical University Hospital) and was registered in UMIN (ID: 000036503). This study was reported according to the STROBE guidelines [9] and all of the methods described herein were conducted in accordance with the relevant guidelines and regulations. The present study was conducted in accordance with the Declaration of Helsinki and informed consent was obtained from all patients.

### 2.2. Patient Population

Consecutive patients, up to 25 in each participating hospital, who stayed in the ICU for more than 48 h between June and December in 2019 were eligible for enrollment. The present study was conducted at nine ICUs, which were mixed ICUs including surgical, medical, and emergency ICUs. Patients younger than 18 years of age, unable to walk independently before admission, with neurological complications (stroke, severe head injury, central nervous system infection, brain tumor, neurosurgery, post-cardiopulmonary resuscitation with hypoxic encephalopathy, cervicobrachial injury with consciousness impairment, cerebrovascular dementia, Alzheimer’s disease, Parkinson’s disease, etc.), lacking communication skills due to pre-existing mental diseases, or in a terminal state were excluded. The decision regarding the above exclusions was made by the attending physician at each facility.

### 2.3. Data Collection

The following data were collected from the medical records of each patient. Baseline characteristics were collected at the time of ICU admission and during ICU stays by co-investigators at each hospital, including age, sex, body mass index, the Charlson comorbidity index [10], the Barthel Index before hospitalization [11], the reason for ICU admission, Acute Physiology and Chronic Health Evaluation II (APACHE II) scores, Sequential Organ Failure Assessment scores, and the use of mechanical ventilation, continuous vasopressors, continuous analgesia, continuous sedation, and dialysis. The Barthel index before hospitalization was scored at the time of ICU admission based on information obtained from family members or patients if they were conscious. The average sedation level, described according to the Richmond Agitation-Sedation Scale, was calculated based on data in electronic medical records.

The ICU Mobility Scale (IMS), type of rehabilitation, duration of rehabilitation, and any treatment or medical equipment during rehabilitation were prospectively evaluated for each rehabilitation session at the ICU bedside. The times from ICU admission to the initiation of the rehabilitation program, sitting on the edge of the bed, standing, and walking were recorded. IMS provides a sensitive 11-point ordinal scale ranging from no activity (lying/passive exercises in bed, score of 0) to independent ambulation (score of 10) [12]. In addition, in the present study, “mobilization” was defined as “sitting on the edge of the bed/standing/walking of IMS level three or higher”. If mobilization was not performed, the reason was surveyed at the bedside, on a daily basis and in a systematically evaluated and open-ended format, as barriers to the implementation of mobilization.

Adverse events of hypertension, hypotension, sinus tachycardia, sinus bradycardia, desaturation, tachypnea, hypopnea, arrhythmia, unplanned catheter removal, a fall, and death/cardiac arrest for each rehabilitation session were prospectively and systematically assessed. Hypertension and hypotension were defined as a systolic blood pressure of <80 or >200 mmHg and a Mean Arterial Pressure of <55 or >140 mmHg, respectively, or as a 20% change from the baseline. Sinus tachycardia/bradycardia was defined as a heart rate of <40 or >130 cycles/min, respectively. Desaturation was defined as SpO_2_ < 80% or a 10% decrease from the baseline. Tachypnea/hypopnea was defined as a respiratory rate of <5 or >40 breaths/min, respectively.

### 2.4. Statistical Analysis

Data are presented as a mean with the standard deviation (SD), a median with the interquartile range (IQR), or as a number with a percentage. The *t*-test was used to analyze continuous variables, the Mann–Whitney U test to analyze ordinal scale variables, and the χ^2^ test or Fisher’s exact test for nominal variables, where appropriate. A comparison of characteristics between the two groups of patients mobilized during their rehabilitation sessions and those who were not was conducted. Sample sizes were not selected a priori due to the exploratory nature of this study. All analyses were performed using Stata version 17.0 (Stata Corp, College Station, TX, USA). Statistical tests were two-sided, and the significance of differences was defined as a *p*-value < 0.05. Missing data were excluded.

Open-ended responses related to barriers to the implementation of early mobilization were analyzed using a thematic analysis [13]. Reasons for not mobilizing, described in an open-ended form, were annotated, and coded based on their content. We identified a common theme among the barriers listed. Data were summarized in a table and cross referenced. Microsoft Excel software version 16.73 (23051401) was used to conduct the data analysis. We supplemented these thematic analyses with aspects of trustworthiness strategies, such as verifying data accuracy, peer debriefing, and keeping an audit trail [13]. In addition, the frequency and percentage of each common theme were described.

## 3. Results

### 3.1. Baseline Characteristics

**Figure 1:**
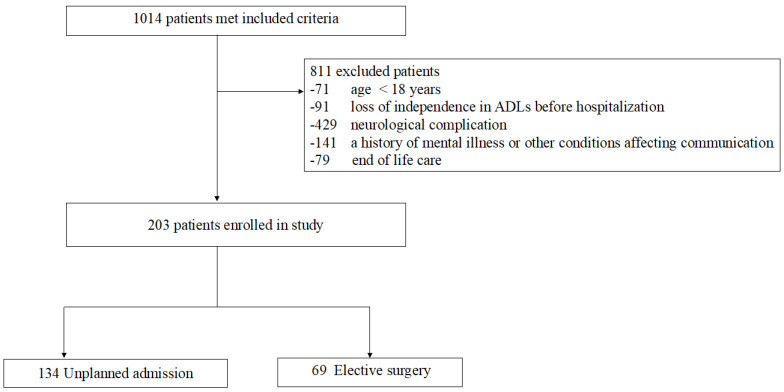
Flow chart of patient recruitment. ADL: activities of daily living.

The 203 patients enrolled in the present study were divided into 69 elective surgical patients and 134 unscheduled admission patients (Figure 1). Patient characteristics are summarized in Table 1. The mean age of patients was 70.2 (SD 15.5) in the unplanned admission group and 65.3 (12.1) in the elective surgery group. APACHE II scores were 21.1 (9.4) and 18.0 (5.7), respectively.

### 3.2. Rehabilitation during the ICU Stay

During the ICU stay, 1049 rehabilitation sessions were conducted in the unplanned admission group and 386 in the elective surgery group (Table 2). Mean times to rehabilitation were 2.9 (7.7) and 1.7 (2.0) days from ICU admission, respectively. Median IMS were five (IQR three and eight) and six (IQR three and nine), respectively. Most patients in both groups were mobilized in a standing position during the ICU stay.

**Figure 2:**
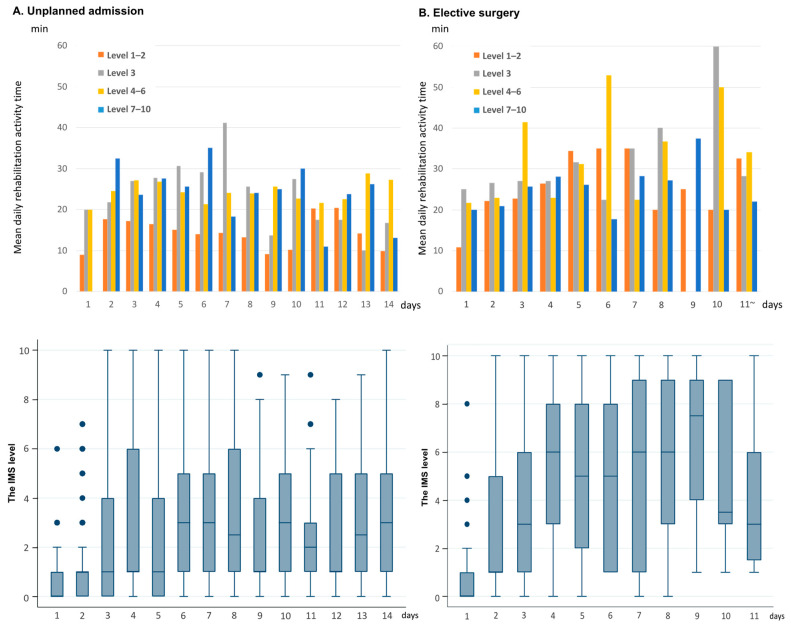
Daily rehabilitation activity time at each IMS level and the median IMS level for each day. IMS; Intensive Care Unit Mobility Scale. The figure shows the daily rehabilitation activity time for each IMS level (top) and the median IMS level for each day (bottom). If a patient is unable to move and is lying in bed, the score is 0. When a patient is able to walk independently without a gait aid, the score is 10.

**Figure 3:**
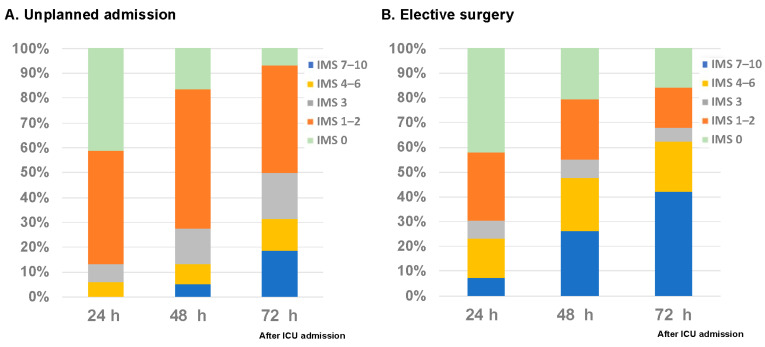
Implementation rate of rehabilitation at each IMS level within 72 h after ICU admission. IMS; Intensive care unit mobility scale. The rate of rehabilitation at each IMS level within 72 h is shown. If a patient is unable to move and is lying in bed, the score is 0. When a patient is able to walk independently without a gait aid, the score is 10.

The duration of daily rehabilitation activity at each IMS level and the median IMS level for each day were shown in Figure 2. The rehabilitation time at a higher IMS level increased up to the third day of ICU admission, whereas no marked changes were observed with time after the fourth day. In addition, median IMS levels increased until day four, after which no marked changes were observed over the days. Figure 3 shows the rate of rehabilitation at each IMS level within 72 h after ICU admission. After 72 h, 50% of unscheduled ICU patients (67/134) continued to receive rehabilitation in bed; however, the percentage of mobilized patients subsequently increased with time. On the other hand, 68.1% (47/69) of postoperative patients were mobilized at level three or higher by day three, 42% (29/47) of whom were walking.

### 3.3. Barriers to and Safety Events in the Implementation of Mobilization in the ICU

The characteristics of patients during rehabilitation sessions who were not mobilized at IMS level three or higher in the ICU are shown in Table 3. In addition, Table 3 shows the safety events for unscheduled ICU admission cases and elective surgery cases. Patients who did not mobilize to a sitting position or higher were more likely to receive ventilation (50.2% vs. 25.3%, *p* < 0.001 in the unplanned admission group; 62.4% vs. 11.0%, *p* < 0.001 in the elective surgery group). In addition, they were more likely to be administered vasopressor agents (43.8% vs. 22.2%, *p* < 0.001 in the unplanned admission group; 55.9% vs. 21.5%, *p* < 0.001 in the elective surgery group). A total of 135 physiological abnormalities and potential safety events occurred, representing 9.4% of 1435 physical therapy sessions.

The most common barriers to performing mobilization in the ICU by a content analysis were circulatory instability (29.9%), followed by catheterization of the femoral artery/vein (15.9%), and coma or deep sedation (13.4%) in the unplanned admission group (Table 4). On the other hand, in the elective surgery group, the most common barriers to performing mobilization were a physician’s order for postoperative bed rest (23.4%), circulatory instability (14.7%), and catheterization of the femoral artery/vein (12.4%). The median (IQR) dose of noradrenaline in rehabilitation sessions when an unstable circulatory status was considered a barrier was 0.1 µg/kg/min (0.04–0.21) in the unplanned admission group and 0.03 µg/kg/min (0.0–0.06) in the elective surgery group.

## 4. Discussion

The present study described the current practice and barriers to the implementation of mobilization in ICUs. The initial results obtained suggested that rehabilitation programs were initiated later for patients whose admittance was unplanned, had a shorter duration, and was less intense. Furthermore, the intensity and duration of rehabilitation for critically ill patients did not increase with time after ICU admission. On the other hand, mobilization was performed even during the use of vasopressors and/or a ventilator, and only a few adverse events were reported. The most common barrier to mobilization was an unstable circulatory status in the unplanned admission group, whereas it was physician orders in the elective surgery group.

Patients with unscheduled ICU admissions may not be able to begin a rehabilitation program immediately or increase the intensity of rehabilitation due to their critical illness. In previous studies on mobilization interventions, only approximately 30–40% of ventilated patients achieved out-of-bed mobilization [14,15]. Moreover, critically ill patients are vulnerable to a number of complications, such as muscle weakness, joint stiffness, and a loss of mobility due to prolonged bed rest, and the time elapsed does not contribute to improvements in rehabilitation intensity or duration, as demonstrated in the present study. Therefore, severely ill patients may need rehabilitation strategies other than waiting for recovery from the disease. Multiple daily rehabilitation sessions, even if only for a short period of time, may be beneficial for these patients to quickly recover muscle strength and mobility [16]. In addition, surgical patients who are admitted in ICU for a long period may have some kind of complication. In the present results, the percentage of rehabilitation intensity differed after day nine. This may indicate that the patient would have different characteristics from those who had left the ICU by day eight, although this is not clear in the present study. Therefore, future research on the characteristics and the effective rehabilitation of these long-term admitted surgical ICU patients may be needed.

The present study reported a small number of adverse events associated with the implementation of mobilization in critically ill patients. A meta-analysis recently showed a pooled safety event rate of 3.2% and a frequency of potential safety events in individual studies ranging between 0 and 23% [17]. The safety event rate was expected to be similar to that in the present study. Adverse events commonly occur in the ICU, even without patient mobility/rehabilitation, with a reported adverse event frequency of >37% during morning care in the ICU [18]. The incidence of safety events in the present study was low, and, thus, rehabilitation may have been performed too cautiously. In addition, the rate of safety events in the present study was not higher in the unplanned admission group than in the elective surgery group. This result suggests increasing the intensity and duration of rehabilitation for unplanned admission patients to the same levels as those for elective surgery patients.

Perceived barriers were mainly related to patient factors, most often hemodynamic and medical instabilities, which was consistent with previous findings [19,20,21]. An unstable circulatory status was the most frequent barrier to rehabilitation in the unplanned admission group. Previous studies reported that mobilization with norepinephrine did not affect mortality and did not lead to a significant increase in adverse events at higher doses of norepinephrine than those at lower doses. A previous study confirmed that mobilization was safe with doses of norepinephrine up to 0.20 µg/kg/min for out-of-bed mobilization and 0.33 µg/kg/min for in-bed mobilization [22]. However, patients in the unplanned admission group with a noradrenaline dosage of about 0.1 µg/kg/min (0.04 to 0.21) were considered unable to perform rehabilitation. The medical practice may consider even this level of catecholamine a barrier, and may not consider its use to count as performed rehabilitation. They may consider even lower catecholamine doses to be a barrier in elective surgical patients and may be more prudent.

### 4.1. Clinical Implication

Conditions for initiation criteria in the mobilization protocol may be revised. In particular, we should reconsider the barriers related to circulatory conditions. This might help achieve early mobilization even in emergency admissions. in elective surgery patients, or in lower doses of catecholamines.

### 4.2. Study Limitations

There are several limitations that need to be addressed. Since this was an observational study, causal relationships remain unclear. Furthermore, the present results may have limited generalizability because this study could not achieve the planned sample size and the number of patients examined was not large. Moreover, although we analyzed data on mobilization, we were unable to examine the clinical course of each disease in all patients. In addition, whether the patient could receive rehabilitation at the level of sitting on the edge of the bed or higher depended on the rehabilitation policy used in each participating hospital. Therefore, whether EM could not be provided due to poor general conditions or other factors was not identified. In the analysis on barriers to rehabilitation and safety events, repeated data collection during the ICU may have biased the results, as some patient characteristics appeared multiple times in the analysis. However, repeated rehabilitation session data during the ICU stay can provide real data on rehabilitation sessions in the ICU.

## 5. Conclusions

Rehabilitation programs were initiated later for unplanned admitted patients and were less intense than those for elective surgical patients, irrespective of the time after ICU admission. The most common barrier to mobilization was an unstable circulatory status in the unplanned admission group, whereas it was physician orders in the elective surgery group.

## Figures and Tables

**Table 1:** Patient characteristics.

	Unplanned Admission n = 134	Elective Surgery n = 69	*p* Value
Age, year, median (IQR)	74.5 (62.3, 81.0)	68.0 (57.0, 74.0)	0.023
Sex, Female, n (%)	83 (61.9)	48 (69.6)	0.353
BMI, median (IQR)	22.3 (20.0, 24.9)	23.5 (21.0, 26.2)	0.118
Reason for ICU admission, n (%)			<0.001
Cardiovascular disease	41 (30.6)	39 (56.5)	
Respiratory disease	33 (24.6)	5 (7.3)	
Abdominal/gastrointestinal disease	19 (14.2)	21 (30.4)	
Sepsis	28 (20.9)	0 (0)	
Renal/metabolic disease	7 (5.2)	0 (0)	
Other	6 (4.5)	4 (5.8)	
Charlson Comorbidity Index, median (IQR)	2.0 (0.25, 3.0)	2.0 (1.0, 3.0)	0.442
Barthel Index prior to hospital admission, mean (SD)	96.6 (9.9)	97.4 (9.3)	0.176
APACHE II score, median (IQR)	20.0 (14.3, 26.8)	18.0 (14.0, 22.0)	0.03
SOFA at ICU admission, median (IQR)	7.3 (9.4)	7.0 (5.0, 10.0)	0.593
Mechanical ventilation, n (%)	81 (60.5)	47 (68.1)	0.221
Ventilator days, day, median (IQR)	1.9 (0, 5.4)	1.0 (0, 2.0)	0.167
Length of ICU stay, day, median (IQR)	5.0 (4.0, 9.0)	4.5 (3.0, 6.4)	0.016
Length of hospital stay, day, median (IQR)	24.0 (15.0, 45.0)	28.0 (21.0, 51.1)	0.02
ICU death, n (%)	10 (7.5)	0 (0)	0.017
Hospital death, n (%)	25 (18.7)	0 (0)	<0.001

BMI; Body Mass Index, APACHE II score; Acute Physiology and Chronic Health Evaluation II score, SOFA: sequential organ failure assessment.

**Table 2:** Rehabilitation during the ICU stay.

During the ICU Stay	Unplanned Admission n = 134	Elective Surgery n = 69
Time from ICU admission to the start of rehabilitation programs, day, median (IQR) (n = 134)	1.1 (0.7, 2.0)	0.8 (0.5, 2.0)
Time from ICU admission to sitting on the edge of the bed, day, median (IQR) (n = 120)	3.0 (2.0, 5.0)	1.9 (0.9, 4.0)
Time from ICU admission to standing, day, median (IQR) (n = 117)	4.4 (2.6, 6.7)	2.8 (1.5, 6.1)
Time from ICU admission to walking, day, median (IQR) (n = 107)	6.0 (3.4, 10.1)	4.0 (2.7, 7.9)
Max IMS, Median (IQR)	5 (3, 8)	6 (3, 9)
Total rehabilitation sessions	1049 sessions	386 sessions
Rehabilitation activity time, min, mean ± SD	19.4 ± 17.3	26.4 ± 19.0
IMS level 3, n (%)	446 (42.5)	200 (51.8)
ROM, n (%)	476 (45.3)	114 (29.5)
EMS, n (%)	162 (15.4)	6 (1.6)
Respiratory physiotherapy, n (%)	283 (27.0)	101 (26.2)
Sitting on the edge of the bed, n (%)	160 (15.3)	46 (11.9)
Standing, n (%)	166 (15.8)	68 (17.6)
Waking, n (%)	122 (11.6)	88 (22.8)

IQR; Interquartile range, IMS; Intensive Care Unit Mobility Scale, ROM; Range of Motion, EMS; Electrical Muscle Stimulation.

**Table 3:** Characteristics of patients who did not mobilize at IMS level three or higher during each rehabilitation session.

	Unplanned Admission		Elective Surgery	
	Mobilized at IMS Level 3 or Higher		Mobilized at IMS Level 3 or Higher	
	No, 603 Sessions	Yes, 446 Sessions	No, 186 Sessions	Yes, 200 Sessions
IPPV/NPPV, n (%)	303 (50.2)	113 (25.3)	116 (62.4)	22 (11.0)
Vasopressor, n (%)	264 (43.8)	99 (22.2)	104 (55.9)	43 (21.5)
CHDF, n (%)	94 (15.6)	35 (7.8)	23 (14.1)	1 (0.5)
Sedation, n (%)	256 (42.5)	93 (20.9)	88 (47.3)	19 (9.5)
Analgesia, n (%)	313 (51.9)	151 (33.9)	114 (61.3)	75 (37.5)
Delirium, n (%)	89 (14.8)	53 (11.9)	26 (14.1)	14 (7.0)
RASS before rehabilitation, Median (IQR) *	−1 (−2, 0)	0 (0, 0)	0 (−1, 0)	0 (0, 0)
Physical restraint before rehabilitation, n (%) *	244 (40.7)	139 (31.2)	90 (50.6)	5 (2.5)
Total safety events	21 (3.5)	77 (17.3)	7 (3.8)	26 (13.0)
Hyper/hypotension ^†^	9 (1.5)	39 (8.7)	5 (2.6)	26 (13.0)
Sinus tachy/bradycardia ^‡^	2 (0.3)	14 (3.1)	0 (0)	0 (0)
Arrhythmia	0 (0)	1 (0.2)	0 (0)	0 (0)
Desaturation ^§^	7 (1.2)	13 (2.9)	2 (1.0)	0 (0)
Tachy/Hypopnea ^||^	3 (0.5)	4 (1.3)	0 (0)	0 (0)
Unplanned catheter removal	0 (0)	6 (1.3)	0 (0)	0 (0)
A fall	0 (0)	0 (0)	0 (0)	0 (0)
Death	0 (0)	0 (0)	0 (0)	0 (0)

IMS; Intensive care unit mobility scale, IPPV; intermittent positive pressure ventilation, NPPV; non-invasive positive pressure ventilation, CHDF; continuous hemodiafiltration, ECMO; extracorporeal membrane oxygenation, RASS; Richmond Agitation-Sedation Scale. * The sample size was 1212 in the unplanned admission group and 375 in the elective surgery group because only IMS level one or higher was represented. ^†^ Hypertension and hypotension were defined as a systolic blood pressure of <80 or 200 mmHg and MAP <55 or 140 mmHg, respectively, and a 20% change from the baseline. ^‡^ Sinus tachycardia/bradycardia was defined as a heart rate of <40 or 130 cycles/min, respectively. ^§^ Desaturation was defined as SpO_2_ < 80% or a 10% decrease from the baseline. ^||^ Tachypnea/hypopnea was defined as a respiratory rate of <5 or 40 breaths/min, respectively.

**Table 4:** Barriers to the implementation of mobilization in the ICU by a thematic analysis.

Reason	Unplanned Admission	Elective Surgery
	Number of Respondents = 930, n (%)	Number of Respondents = 218, n (%)
Unstable circulatory status	278 (29.9)	32 (14.7)
Catheterization of the femoral artery/vein ^†^	148 (15.9)	27 (12.4)
Coma/deep sedation	125 (13.4)	23 (10.6)
Unstable respiratory status	106 (11.4)	24 (11.0)
Specific diseases requiring bed rest *	69 (7.4)	1 (0.5)
Inadequate staffing/excessive workloads	65 (7.0)	11 (5.1)
Patient symptoms (painful/dyspnea/ fatigue)	35 (3.8)	20 (9.17)
Laboratory tests and/or procedures	21 (2.3)	8 (3.7)
Extreme musculoskeletal weakness	13 (1.4)	3 (1.4)
Delirium/agitation	11 (1.2)	5 (2.3)
A postoperative bed rest order by a physician	0 (0)	51 (23.4)
Unclear reason	59 (6.3)	13 (6.0)

Multiple answers available. * Included post percutaneous coronary interventions in acute coronary syndrome/conservative therapy for acute aortic dissection/active bleeding. ^†^ Included extracorporeal membrane oxygenation/intra-aortic balloon pumping/continuous hemodiafiltration.

## Data Availability

The dataset generated and analyzed during the present study is not publicly available, but is available from the corresponding author upon reasonable request.

## Abbreviations

ICU	intensive care units
APACHE II score	Acute Physiology and Chronic Health Evaluation II score
IMS	ICU Mobility Scale
SD	standard deviation
IQR	interquartile range
